# Self-propelled Leidenfrost droplets on a heated glycerol pool

**DOI:** 10.1038/s41598-021-83517-1

**Published:** 2021-02-17

**Authors:** Ryo Matsumoto, Koji Hasegawa

**Affiliations:** 1 Graduate School of Engineering, Kogakuin University, Tokyo, Japan; 2 Department of Mechanical Engineering, Kogakuin University, Tokyo, Japan

**Keywords:** Fluid dynamics, Mechanical engineering, Thermodynamics

## Abstract

The development of contactless sample manipulation for microfluidic purposes has attracted significant attention within the physicochemical fields. Most existing studies focus on the interactions of unheated liquid substrates and on heated/unheated solid substrates. Therefore, the dynamics of droplets on heated liquid pools have yet to be explored. Here, we present an experimental investigation on the levitated and self-propelled droplets on a heated pool. We aim to identify the effect of the pool temperature and the thermophysical properties of droplets on the dynamics of a self-propelled Leidenfrost droplet on a heated pool. The motion of droplets after levitation on the heated pool is visualized. To elucidate the self-propulsion of Leidenfrost droplets, we quantify the thickness of the vapour film between the approaching droplet and the pool surface. Our experimental results show a quantitative agreement with the simple model prediction for self-propelled Leidenfrost droplets. Our results provide deeper physical insights into the dynamics of Leidenfrost droplets on a heated pool for contactless and contamination-free sample manipulation.

## Introduction

Droplet impact on liquid^1–3^ and solid^4–6^ substrates is ubiquitous in nature and industry. When a low boiling point fluid is dropped onto a sufficiently heated substrate, the droplets do not make direct contact, but levitate on the heated substrate^7–15^. This levitation phenomenon, especially for heated solid substrates, is known as the Leidenfrost effect^16^. As a droplet approaches a heated substrate, its vapours act as a cushion between itself and the substrate. Although many descriptions have been provided for the Leidenfrost effect with respect to droplets on heated solid substrates, the underlying physical phenomena for droplets approaching heated liquid substrates (i.e., pools) is less studied because of the complexity of the dynamics involved. Deciphering the underlying dynamics of the Leidenfrost effect for heated pools has potential benefits for diverse scientific applications, specifically the development of contactless microfluidic devices^17,18^. As such, the Leidenfrost effect is highly relevant for a wide range of fields, including fundamental thermo-fluid dynamics, spray cooling, combustion process, and even aerosol science^2,5,6,19^.

Several efforts have been made to provide a better understanding of the essential physics of Leidenfrost droplets on a heated pool^7–11,20,21^. Davanlou^7^ studied how the physical properties of droplets affect their lifetime when suspended on a liquid pool using a lubrication theory. Maquet et al.^8^ demonstrated that the Leidenfrost effect can arise even when the surface temperature only slightly exceeds the boiling point of the droplet. Additionally, Janssens et al.^9^ visually demonstrated that acetone droplets can be levitated and self-propelled on a heated surface. Kirar et al.^10^ investigated the behaviour of ethanol droplets on a liquid surface, and showed how the partial coalescence of droplets on a liquid surface varies with droplet size and the drop height from the liquid pool. Furthermore, Savino et al.^20^ showed that the pressure required to support droplets on a liquid surface is generated by the thermal capillary effects created by air flowing into the voids below the droplet. In addition, the interplay between Leidenfrost droplets suspended on a hot pool and the Marangoni convection within the pool has been simulated by Sobac et al.^21^. Recently, the inverse Leidenfrost phenomenon, in which a droplet is lifted by the vapour generated by the evaporation of the liquid surface, has been explored^22–26^. Gauthier et al.^25,26^ experimentally and numerically analysed the asymmetry of the vapour film thickness underneath the droplet and determined the heading direction of self-propelled droplets on a cold liquid nitrogen surface. Additionally, multiple Leidenfrost droplets have been levitated on a liquid nitrogen surface, revealing that the droplet-to-droplet interactions lead to the drops spontaneously orbiting each other^27^. Although different approaches have been adopted to describe the properties of Leidenfrost droplets on liquid surfaces as well as the effects of Marangoni convection^28,29^, the self-propulsion of droplets on liquid surfaces has yet to be explored extensively.

We aim to demonstrate the effect of the pool temperature and the thermophysical properties of droplets on the dynamics of Leidenfrost droplets on a heated pool. Volatile droplets (acetone and ethanol) and a glycerol pool (selected for its high boiling point) were used to identify the physical parameters required for Leidenfrost-induced levitation and self-propulsion on a heated pool. The effects of varying the droplet composition (acetone and ethanol) and the pool surface temperature are investigated with the experiments and theoretical model. To elucidate the physical phenomena of Leidenfrost droplets, the thickness of the vapour film separating the surfaces of the droplet and the pool is predicted. The time evolution of the velocity of ethanol droplets during self-propulsion is quantified and discussed using a kinetic model to analyse the interplay between the propulsion and the viscous drag force. The self-propulsion of Leidenfrost droplets on the heated liquid surface is considered as a means for transporting droplets of various sizes without any contact from the pool itself or the wall enclosing it. These results offer important insights for contactless micro-thermo-fluidic technologies, such as a micromanipulation^13–15^.

## Results and discussion

### Levitation of droplets on a heated pool

Experiments were conducted for acetone and ethanol droplets at *ΔT* = 0, 10, 20, and 30 °C, where *ΔT* is the difference between the surface temperature *T*_*p*_ and the saturation temperature *T*_*sat*_. Table 1 provides a summary of the experimental results. Circles (〇) represent the levitation/self-propulsion conditions and crosses (×) represent the droplet settling on the pool. The diameter of the droplets is approximately 2 mm. The experimental results for *ΔT* ≅ 0 °C (i.e., *T*_*p*_ ≅ *T*_*sat*_) show that the droplets settled rather than levitated. For *T*_*p*_ ≥ *T*_*sat*_, the acetone and ethanol droplets levitated at *ΔT* ≥ 20 °C and *ΔT* ≥ 10 °C, respectively. This suggests that the evaporation-induced vapour cushion between the droplet and pool plays a vital role in the stable levitation of droplets above the heated pool.

**Table 1:** Summary of the droplet levitation/self-propulsion conditions.

Droplet (Boiling point)	*ΔT* (= *T*_*p*_* − T*_*sat*_) [°C]			
	0	10	20	30
Acetone (56 °C)	×	×	〇	〇
Ethanol (78 °C)	×	〇	〇	〇

**Figure 1:**
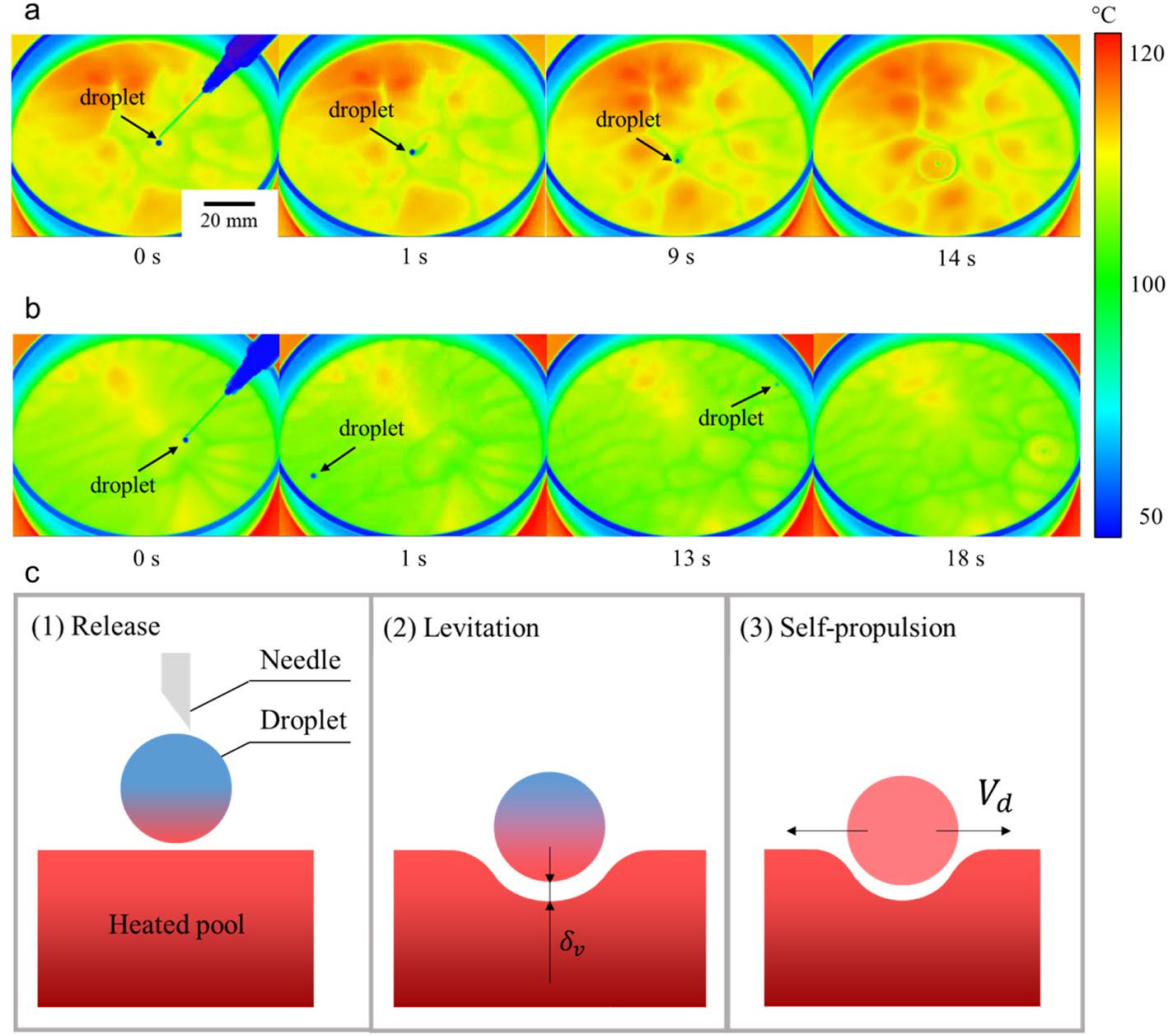
Droplet behaviour on heated pool. Snapshots of the temperature field obtained by an IR camera for (**a**) acetone droplet and (**b**) ethanol droplet. The droplet diameter was 2 mm. The acetone and ethanol droplets settled on the surface of the pool after approximately 14 s and 18 s, respectively. This information is also supported by Supplementary Videos 1 and 2. (**c**) Illustration of the droplet behaviour on the heated pool.

Figure 1 shows the droplet behaviour obtained by the IR camera for *ΔT* ≅ 30 °C. For the acetone droplet shown in Fig. 1a and Supplementary Video 1, the droplet levitated on the liquid pool after the droplet release and was subsequently self-propelled. It then moves around the centre of the vessel before eventually settling on the surface of the pool (14 s after levitation was initiated). The ethanol droplet shown in Fig. 1b and Supplementary Video 2 also demonstrated self-propulsion, colliding repeatedly with the vessel wall. On this occasion, the ethanol droplet settled on the pool surface 18 s after the levitation process began. Based on the experimental results obtained from Fig. 1a,b), the droplet behaviour on the heated pool can be described with the following processes: (1) droplet release, (2) levitation (Leidenfrost effect), and (3) self-propulsion, as illustrated in Fig. 1c.

**Figure 2:**
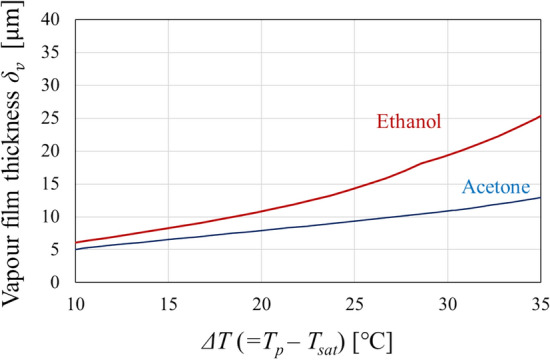
Estimation of vapour film thickness. The vapour film thickness was estimated using Eq. (1) for each droplet liquid. The red and blue curves indicate *δ*_*v*_ for ethanol and acetone, respectively.

For droplet levitation to occur, the vapour film formed via evaporation, as the droplet approaches the pool surface, must be sufficiently thick. The thickness of the vapour film *δ*_*v*_ can be expressed as Eq. (1), which considers the balance between the thermal Marangoni force and the gravitational force on the droplet^7^. Here, lubrication theory is applied to predict the vapour film thickness *δ*_*v*_:

1 $${\delta }_{v}\sim \sqrt{\frac{{\sigma }_{T}{\mu }_{v}}{{\rho }_{d}g{\mu }_{p}}\left({T}_{p}-{T}_{d}\right)},$$

where *σ*_*T*_ is the interfacial tension gradient of the droplet, *ρ*_*d*_ is the density of the droplet, *g* is the gravitational acceleration, *μ*_*v*_ is the viscosity of the vapour from the droplet, *μ*_*p*_ is the viscosity of the pool, *T*_*p*_ is the temperature of the pool, and *T*_*d*_ is the temperature of the droplet surface. The thickness of the vapour film separating the droplet and the pool can be estimated by substituting the physical properties of glycerol (liquid surface), acetone or ethanol (droplets), and the droplet temperature from the experiments into Eq. (1). Figure 2 presents the vapour film thickness prediction curves for acetone (blue curve) and ethanol (red curve). In both cases, the vapour film thickness increases as *ΔT* (= *T*_*p*_ − *T*_*sat*_) increases. As demonstrated in a previous study^7^, the levitation time of the droplets is proportional to *ΔT*. This is due to the vapour film thickness mitigating heating from the pool surface. The estimation curves in Fig. 2 indicate that the ethanol vapour film is thicker than its acetone counterpart. This is responsible for the low friction at the interface of the ethanol droplet and provides an explanation for the faster motion of the droplet in Fig. 1b and Supplementary Video 2.

### Self-propulsion of droplets after levitation

**Figure 3:**
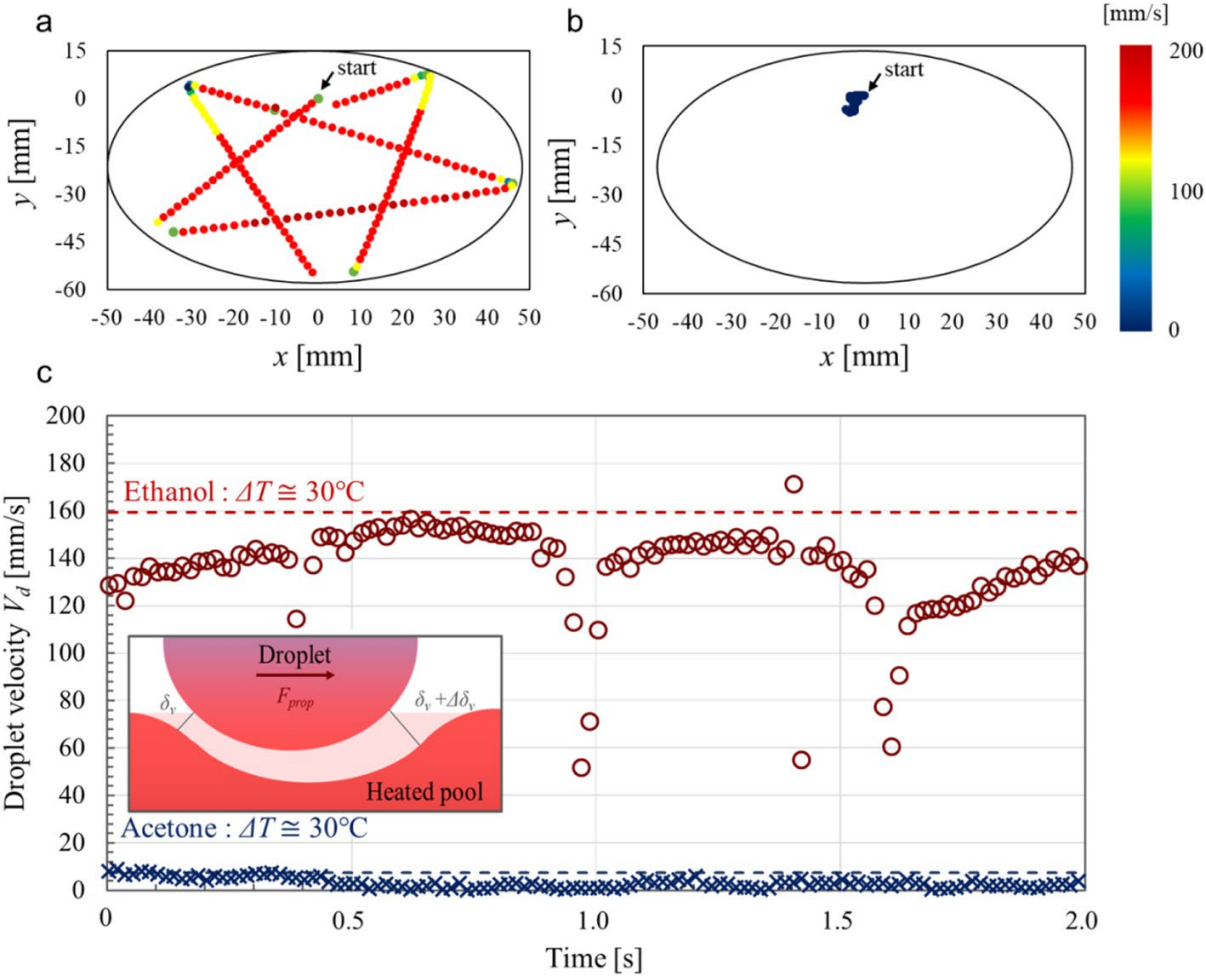
Self-propulsion of ethanol and acetone droplets. The trajectory of the droplets at a given time is shown. The initial position of the droplets is represented by the origin. (**a**) Ethanol droplet with *ΔT* ≅ 30 °C. The trajectory represents the first 3 s of droplet motion. (**b**) Acetone droplet with *ΔT* ≅ 30 °C. The trajectory represents the entire droplet motion over a duration of 14 s. (**c**) Comparisons of the experimental data against the model prediction of droplet self-propulsion. The red circles (**〇**) and blue crosses (**×**) represent the experimental results for the ethanol and acetone droplets, respectively. The terminal velocities *V*^*t*^_*d*_ predicted using Eq. (5) are represented as dashed lines (red for ethanol, blue for acetone). The inset indicates the model for the self-propulsion of the droplet.

Figure 3 presents the self-propulsion characteristics of the Leidenfrost droplets for *ΔT* ≅ 30 °C based on the experimental data in Fig. 1. In Fig. 3a,b, the initial position of the droplet is represented by the origin. The ethanol droplets (Fig. 3a) traversed a star-shaped trajectory as the droplets collide repeatedly with the vessel wall after levitation. As for the velocity, the droplet accelerates after the levitation process, before decelerating drastically as it approaches the vessel wall, then accelerates again immediately after rebounding off the vessel wall. Conversely, the acetone droplet (Fig. 3b) moves irregularly around the centre of the pool surface without colliding with the vessel wall.

To understand the physical mechanism of the self-propulsion of the Leidenfrost droplets on the heated pool in greater detail, we described the droplet motion using a simple model. This model is based on the equation of motion that assumes that the propulsive force of the droplet can be balanced by the viscous drag force on the droplet in a steady state, which is expressed as

2 $$m\frac{d{V}_{d}}{dt}={F}_{prop}+{F}_{D},$$

where *m* is the mass of the droplet, *V*_*d*_ is the droplet velocity, and *t* denotes time. The left-hand side of Eq. (2) describes the inertia force *mdV*_*d*_*/dt.* The first term on the right-hand side of Eq. (2) represents the driving force *F*_*prop*_ of the droplet, which is expressed as

3 $${F}_{prop}\sim {\rho }_{d}g{R}^{2}\Delta {\delta }_{v},$$

where *R* is the radius of the droplet and *∆δ*_*v*_ is the difference in the vapour film thickness between the front and back surfaces of the droplets^25^. The droplets are propelled in one direction owing to the asymmetry of this difference. In addition, we assumed that *∆δ*_*v*_ can be in the order of 1 μm, which is approximated by Gauthier et al.^25^. The second term on the right-hand side of Eq. (2) represents the viscous drag force *F*_*D*_ associated with the propulsion of the droplet, which is proportional to the droplet velocity *V*_*d*_ and inversely proportional to the vapour film thickness *δ*_*v*_ obtained by Eq. (1):

4 $${F}_{D}\sim -{\mu }_{v}\frac{{V}_{d}}{{\delta }_{v}}{R}^{2}.$$

Therefore, we expect the frictional force to increase as the droplet accelerates and the thickness of the vapour film to become thinner over time as *ΔT* decreases. From Eq. (2), we can derive the terminal velocity of the droplet *V*^*t*^_*d*_ by assuming that Eqs. (3) and (4) are balanced after sufficient time has elapsed:

5 $${V}_{d}^{t}\sim \frac{{\rho }_{d}g}{{\mu }_{v}}\Delta {\delta }_{v}{\delta }_{v}.$$

Figure 3c compares the self-propulsion velocities of the Leidenfrost droplets obtained from the experimental data with the terminal velocity *V*^*t*^_*d*_ predicted by Eq. (5). The ethanol droplet accelerates and decelerates periodically (roughly every 0.5 s) between collisions with the vessel wall. The ethanol droplet reached a maximum velocity of approximately 160 mm/s when the droplet crossed the centre of the liquid surface, which represents an excellent agreement with the predicted velocity (also 160 mm/s). For acetone, the measured maximum velocity and the corresponding model prediction were approximately 8 mm/s and 7 mm/s, respectively. As illustrated by Fig. 3c, our experimental results for ethanol and acetone droplets show a quantitative agreement with the model prediction based on Eq. (5). Although the size of the self-propelled droplet decreased with its evaporation, the terminal velocity of the droplet is not a function of the droplet size, as presented in Eq. (5). This is due to the fact that the driving force *F*_*prop*_ of the droplet as shown in Eq. (3) changes with the droplet size, and the following viscous drag force *F*_*D*_ on the droplet, as shown in Eq. (4), also changes. Therefore, the terminal droplet velocity can be independent of the droplet size (see also Supplementary Information). Overall, this simple model successfully captures the essential dynamics of the self-propelled Leidenfrost droplets.

As demonstrated through the results, we have identified the levitation condition and physical mechanism of the self-propulsion of Leidenfrost droplets on a heated pool. However, several challenges still remain in attaining a complete understanding of the phenomena. According to our model, the asymmetry of the vapor film thickness beneath the droplet was predicted in the order of micrometres. This slight asymmetry of vapor film can determine the direction of the droplet. This symmetry breakup can be caused by the slight deformation of the liquid pool surface, and by the approaching droplet. Controlling the direction of motion of the droplet by the designed container and the active temperature gradient needs to be explored.

Subsequently, we discuss the difference of self-propulsion dynamics of the acetone and ethanol droplets. Based on our model, as expressed in Eq. (5), the vapour film thickness *δ*_*v*_ has a great influence for the droplet motion. This vapour film thickness can be determined by the physicochemical properties of the droplet and pool as shown in Eq. (1). Additionally, Eq. (1) also shows that the surface tension gradient and the viscosity ratio of the vapour of the droplet and the pool can affect the vapour film thickness. Due to the effect of the physicochemical properties, the ethanol droplet has a thicker vapour film thickness than that of the acetone. Although we estimated the vapour film thickness with a simple model, achieving a direct and precise visualisation of the spatiotemporal evolution of the vapour film thickness *δ*_*v*_ and its variation *∆δ*_*v*_ is challenging and beyond the scope of the present work. We hope that this study inspires further simulations and experiments that help to elucidate the microfluidics, Marangoni stresses, heat transfer, and evaporation involved in futuristic lab-on-a-drop applications^13–15,17,18^.

## Methods

### Experimental setup

**Figure 4:**
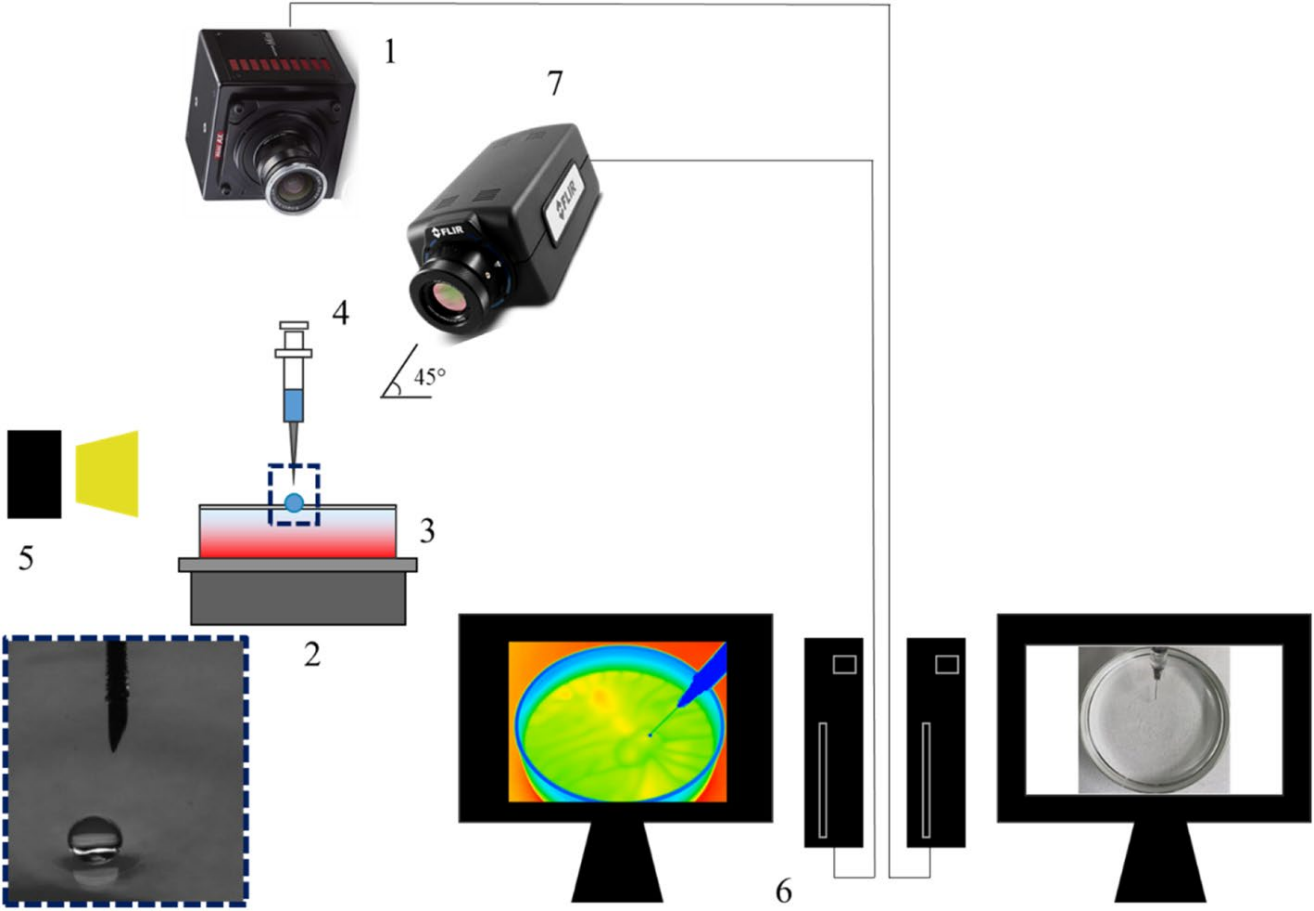
Illustration of the experimental setup. (1) Digital video camera, (2) heater (hot plate), (3) glass vessel, (4) syringe, (5) LED light, (6) computer, and (7) IR camera. Inset picture represents the droplet of diameter 2.0 mm (and needle tip), immediately after the levitation on the heated pool.

Figure 4 illustrates the experimental setup used in the present study. A glass vessel with an internal diameter of 100 mm and a height of 30 mm was used for the experiments, and the depth of the pool *H* was maintained at 10 mm. The experiments were conducted using volatile droplets and a low volatility pool to facilitate the occurrence of Leidenfrost droplets on the heated pool by promoting droplet evaporation while avoiding evaporation from the pool. The boiling points of acetone, ethanol (droplets), and glycerol (pool) at atmospheric pressure are 56, 78, and 290 °C, respectively. The temperature differences *ΔT* between the pool temperature *T*_*p*_ and the saturation temperature (boiling point) of the droplet *T*_*sat*_ were approximately 0, 10, 20, and 30 °C. Although the absolute value of temperature has a key role in influencing the thermophysical properties of the droplet and pool, for the Leidenfrost effect, the temperature difference *ΔT* is more important than the boiling point of the droplet itself, because the heat transfer can be driven by the temperature difference, and not the absolute temperature of the droplet or pool. Acetone and ethanol have different boiling points (56 and 78 °C, respectively), therefore, the temperature difference mentioned above is suitable for a reasonable comparison regarding the heat transfer from the droplet. Each droplet, with an initial diameter of 2.0 mm, was released manually by a syringe onto the heated pool. The glycerol pool was heated by a hot plate (AS ONE, EHP-250N) through the bottom of the vessel, and the temperature of the pool surface was constantly monitored using a digital thermometer to ensure that the pool surface reached a target temperature and steady state conditions prior to the droplet release. The behaviour of the droplets and the temperature field were captured simultaneously using digital cameras (Photron, FASTCAM Mini AX50 type HS-TT and Apple, iPhone 11) as well as an IR camera (FLIR Systems, FLIR A6750sc MWIR). Quantitative analysis of the captured images was performed using ImageJ software, version 1.52 for Windows (https://imagej.nih.gov/ij/, National Institutes of Health, Maryland, USA).

### Statistical analysis

For visual analysis of the droplet motion, the uncertainty in the droplet diameter was less than 8% for *d* = 2.0 mm with a spatial resolution of ~ 160 μm/pixel. For the temperature field measurements, the uncertainty of the IR camera was ± 2% in the present experimental conditions according to the specifications of the instrument^30^.

## Supplementary Information

Supplementary Information 1.

Supplementary Video 1.

Supplementary Video 2.

Supplementary Video 3.

## Data Availability

The datasets generated during and/or analysed during the current study are available from the corresponding author on reasonable request.
