# Supplementary material for: Self-propelled Leidenfrost droplets on a heated glycerol pool
Source: Sci Rep. 2021 Feb 17;11:3954. doi: 10.1038/s41598-021-83517-1 (PMC7889849; doi:10.1038/s41598-021-83517-1)
Supplement: Supplementary file 1 — Supplementary Information 1. [file 41598_2021_83517_MOESM1_ESM.pdf]

Supplementary information

Self-propelled Leidenfrost droplets on a heated glycerol pool

Authors

Ryo Matsumoto[1] and Koji Hasegawa[2*]

[1]Graduate School of Engineering, Kogakuin University, Tokyo, Japan.

[2]Department of Mechanical Engineering, Kogakuin University, Tokyo, Japan.

[*]kojihasegawa@cc.kogakuin.ac.jp

Supplementary Video 1

This video shows the acetone droplet on the heated glycerol pool captured by an IR camera for $\Delta T \cong 30$ °C. The depth of the pool was 10 mm, and droplet diameter was 2 mm. The acetone droplets settled on the surface of the pool after approximately 14 s.

Supplementary Video 2

This video shows the ethanol droplet on the heated glycerol pool captured by an IR camera for $\Delta T \cong 30$ °C. The depth of the pool was 10 mm, and droplet diameter was 2 mm. The ethanol droplets settled on the surface of the pool after approximately 18 s.

Supplementary Video 3

This video shows the ethanol droplet on the heated glycerol pool captured by an IR camera for $\Delta T \cong 30$ °C. The depth of the pool was 2 mm, and droplet diameter was 2 mm. Compared with Supplementary Video 2, the effect of the pool depth is not quantitatively significant.

Sample combination for droplet and pool

Prior to the present experiment, we have verified the suitable combination of the droplet and pool. As summarized Table S1, we used water for a pool and droplet, and 1-propanol/2-propanol for a droplet. Each experiment was done more than 5 times (10 times for especially acetone and ethanol) to confirm the reproducibility. Circles (○) represent the levitation/self-propulsion conditions and crosses (×) represent the droplet settling on the pool.

When we used water for pool, the pool surface was deformed with increasing the pool temperature T_p by the natural convection and boiling (100 °C). To avoid the disturbance of the pool surface, we chose glycerol as a pool for the higher boiling point (290 °C) as well as economic/safety reasons.

As for a droplet, the boiling point have a key role in the Leidenfrost droplet on the heated pool. As indicated Table S1, acetone and ethanol were selected mainly because of the lower boiling point (saturation temperature T_{sat}). This also means lower the temperature for the pool with the same temperature difference. Therefore, we reported the pre-verified cases (acetone and ethanol droplet on heated glycerol pool) in the manuscript. The absolute value of the pool temperature in each condition of the present study is listed in Table S2.

Table S1. Experimental cases in the present study.

Droplet (Boiling point)	ΔT (= $T_p - T_{sat}$) [°C]			
	0	10	20	30
Acetone (56 °C)	×	×	○	○
Ethanol (78 °C)	×	○	○	○
Water (100 °C)	×	×	×	×
1-propanol (97 °C)	×	×	×	×
2-propanol (82 °C)	×	○	○	○

Table S2. Absolute value of pool temperature for each condition.

Droplet (Boiling point)	$\Delta T \cong$ 10 °C	20 °C	30°C
Acetone (56 °C)	~65 °C	~75 °C	~85 °C
Ethanol (78 °C)	~90 °C	~100 °C	~110 °C

Effect of vessel size, depth and shape

Regarding the size of the vessel, we have conducted the experiments with 100 mm (Supplementary Video 2) and 200 mm in diameter. It was confirmed that there was no significant effect on the droplet behavior. As same as the present data (100 mm in diameter), the droplet accelerates after the levitation, before decelerating drastically as it approaches the vessel wall, then accelerates again immediately after rebounding off the vessel wall. For the larger vessel, the frequency of decreases of the droplet velocity (the frequency of droplet collision with the vessel wall) is higher than smaller vessel. For the larger vessel (200 mm in diameter), the frequency of decreases of the droplet velocity (the frequency of droplet collision with the vessel wall) is lower than smaller vessel.

Regarding the pool depth, the pool depth of 2 mm (Supplementary Video 3), 10 mm (Supplementary Video 2) and 30 mm was investigated, and we found no significant effect on the droplet behavior as well. Although the pool depth affects the heat transfer of the pool up to thermal equilibrium (target pool temperature in each experimental condition), the pool depth does not have a critical role in the droplet behavior after reaching the thermal equilibrium.

We confirmed the effect of the dish size and pool depth. However, the effect of the dish shape needs to be explored and beyond the scope of the present work. According to the previous study[26], the vessel shape can play a promising role for the active motion control of the self-propelled droplet.

Effect of initial droplet size and evaporation

In order to clarify the effect of the initial droplet size, we compare two different initial droplet diameters (2.0 mm and 2.4 mm) for the ethanol droplet with ΔT = 30 °C. Figure S1 is added to represent the effect of droplet size on the velocity of the droplet on the heated pool. Although the droplet lifetime was longer for the larger droplet, droplet velocity was overall the same for different droplet sizes. The size of the self-propelled droplet decreased with its evaporation, as shown in Fig. S2. However, as modelled in Eq. (5), the terminal velocity of the droplet is not relevant to the droplet size. This is because of the fact that the driving force F_{prop} of the droplet in Eq. (3) decrease with the instantaneous droplet radius (cross sectional area), and the viscous drag force F_D on the droplet in Eq. (4) decrease with the instantaneous droplet radius as well. Therefore, the

terminal droplet velocity in Eq. (5) is not dependent of the initial and instantaneous droplet size.

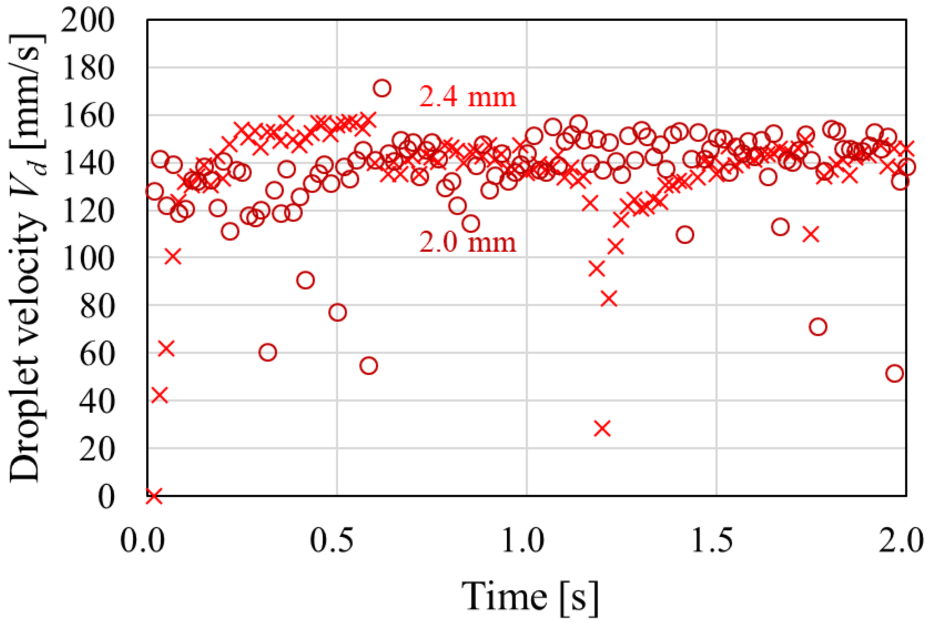

Figure S1. Effect of initial droplet size on the velocity of ethanol droplet for $\Delta T \cong$ 30 °C.

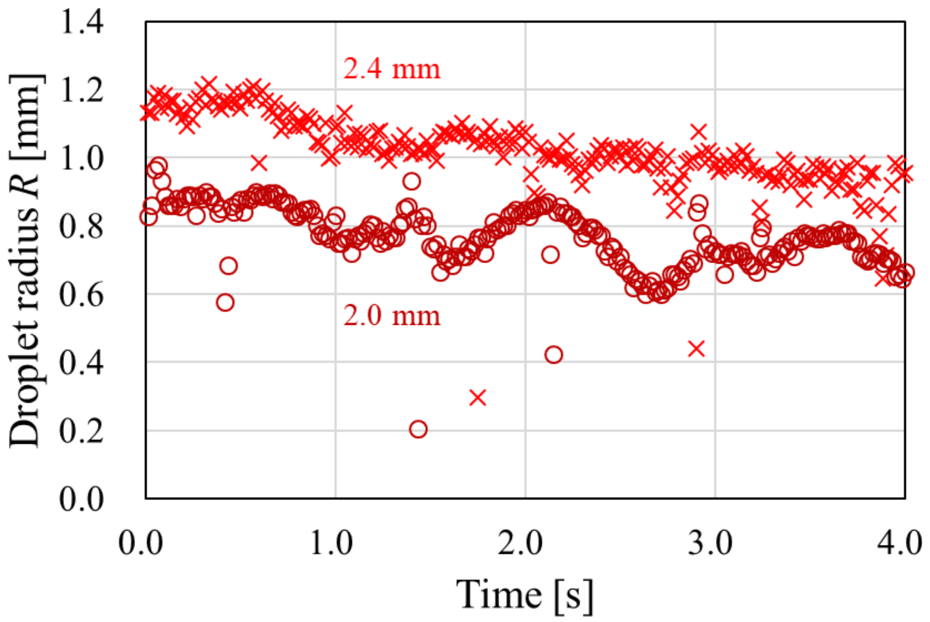

Figure S2. Time volition of the droplet radius for different initial droplet sizes for $\Delta T \cong$ 30 °C.

Effect of temperature difference

Figure S3 represents the effect of the temperature difference ΔT on the droplet velocity of the ethanol. The droplet velocity tends to increase with increasing ΔT. This result is reasonable because a larger ΔT leads to a thicker vapour film, as shown in Eq. (1), which leads to a weaker drag force, in accordance with Eq. (4). Additionally, a thicker vapour film leads to a higher droplet velocity, as predicted by Eq. (5).

Regarding the lifetime of droplets, Fig. S4 represents the effect of temperature difference on the lifetime of the ethanol droplet. The droplet radius fluctuates because the droplets collide repeatedly with the vessel wall after levitation, as shown in Fig. 3 (a). The droplet lifetime slightly increased with increasing ΔT. This can be presumed as a larger ΔT generates a thicker vapour film, in accordance with Eq. (1), which leads to a lower heat transfer from the droplet, as the vapour film beneath the droplet works as a thermal insulator.

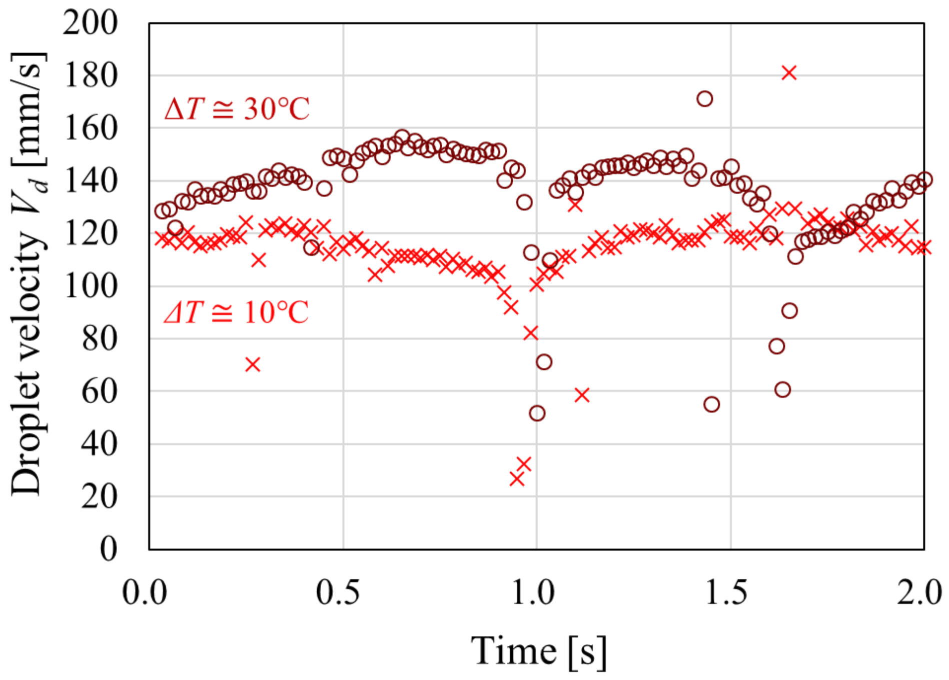

Figure S3. Effect of temperature difference on the velocity of ethanol droplet.

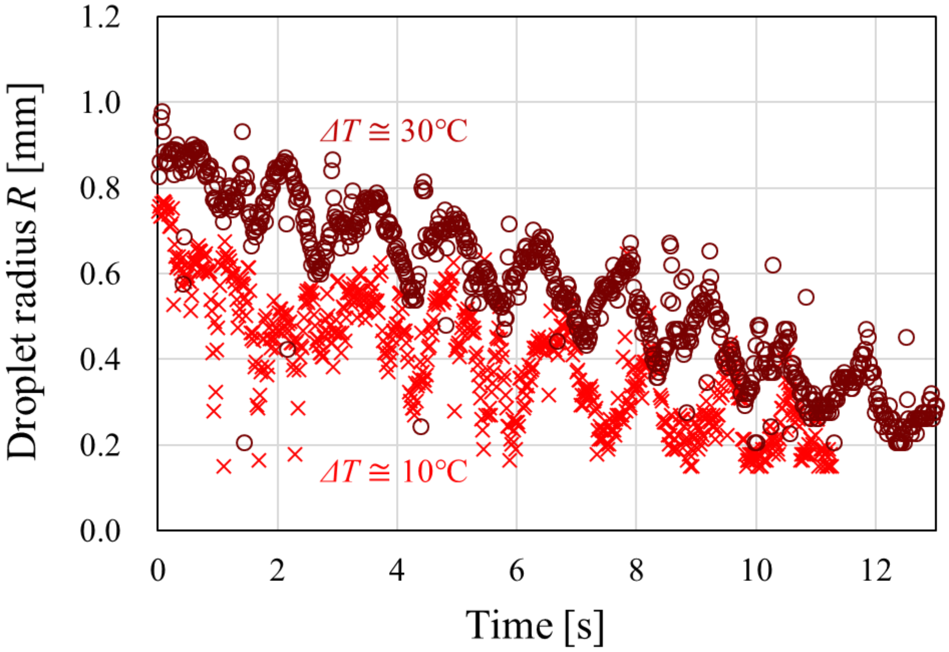

Figure S4. Effect of temperature difference on the lifetime of ethanol droplet.
